# Bringing home the carbon: photorespiratory CO_2_ recovery shows diverse efficiency in *Brassicaceae*

**DOI:** 10.1093/jxb/erad371

**Published:** 2023-11-21

**Authors:** Catherine A Walsh

**Affiliations:** Lancaster Environment Centre, Lancaster University, Lancaster LA1 4YQ, UK

**Keywords:** *Brassicaceae*, carbon-concentrating mechanisms, C_3_–C_4_ intermediates, C_2_ photorespiration, photorespiratory glycine shuttle, photosynthesis

## Abstract

This article comments on:

**Schlüter U, Bouvier JW, Guerreiro R, Malisic M, Kontny C, Westhoff P, Stich B, Weber APM.** 2023. *Brassicaceae* display variation in efficiency of photorespiratory carbon-recapturing mechanisms. Journal of Experimental Botany 74, 6631–6649.

**Photorespiration has long been regarded as a wasteful pathway, expending both carbon and energy. Yet the process is beginning to emerge as an ambiguous pathway, with our understanding not as clear as previously thought. Studies have shown that establishing a photorespiratory pump from the ancestral C**_**3**_ **state to reclaim lost CO**_**2**_ **has been an integral step towards the evolution of C**_**4**_ **photosynthesis. However, not all plant families contain C**_**4**_ **relatives, raising interesting questions about why C**_**4**_ **photosynthesis evolved in some families but not others, and why some species have photosynthetic activity that is neither C**_**3**_ **nor C**_**4**_ **and appears to reiterate intermediate stages that occurred during the evolution of C**_**4**_ **photosynthesis in other families. These plants, termed C**_**3**_ **–C**_**4**_ **intermediates or C**_**2**_ **species, rely on a photorespiratory shuttle to concentrate carbon. Here, Schlüter *et al.* (2023) compile a convincing case for metabolic diversity in the C**_**3**_ **–C**_**4**_ **intermediates and demonstrate convergent evolution within *Brassicaceae* combining anatomical, biochemical, physiological, and genotypic evidence to improve understanding of the C**_**3**_ **–C**_**4**_ **phenotype.**

Photorespiration is the second greatest metabolic flux in the plant, following photosynthesis, occurring when Rubisco fixes O_2_ instead of CO_2_, and is more prevalent under hot, dry conditions when stomata close and limit CO_2_ uptake into the leaf. The oxygenation reaction can account for up to 25% of Rubisco activity, producing the toxic metabolite 2-phosphoglycolate (2PG) which the plant must detoxify using the energy-demanding photorespiratory pathway, subsequently releasing both CO_2_ and NH_3_ in the process (Sharkey, 1988). Previous thinking suggested that photorespiration ran as a closed cycle, yet recent studies indicate that the pathway may partly operate in a non-cyclic manner, interacting with other biochemical pathways to balance cellular stoichiometry in accordance with environmental conditions (Timm *et al.*, 2012; Hodges *et al.*, 2016; Busch, 2020). Consequently, this photorespiratory plasticity may allow variation of biochemical phenotypes both within and between species. In addition, it is now known that photorespiration is not the wasteful pathway once thought, with C_3_ species, including crops such as rice and barley, reclaiming varying degrees of carbon to recoup expenditure (Busch *et al.*, 2018). Photorespiration occurs mainly in C_3_ and C_3_–C_4_ intermediate species. The pathway is largely, but not completely, suppressed by the carbon-concentrating mechanism (CCM) that operates in C_4_ species, thereby favouring carboxylation by Rubisco over oxygenation (Zelitch *et al.*, 2009). It is thought that photorespiration played an intrinsic role in C_4_ evolution, when low atmospheric CO_2_ concentrations 25–30 million years ago and warm environments increased flux through the pathway (Christin *et al.*, 2011; Sage *et al.*, 2018). Under these conditions, acquisition of a photorespiratory pump would improve photosynthetic efficiency and give plants a competitive advantage over those without this CO_2_-concentrating mechanism (Fig. 1). This photorespiratory pump (or C_2_ photosynthesis) is found in C_3_–C_4_ intermediates and optimizes the pathway by splitting the process across two cell types. In these plants, glycine decarboxylase, the enzyme responsible for photorespiratory CO_2_ release, is confined to the bundle sheath cells. This not only increases the path length for photorespiratory CO_2_ to diffuse out of the leaf, thereby increasing the chances of the CO_2_ being recaptured (Rawsthorne *et al.*, 1988), but also raises the concentration of CO_2_ around Rubisco up to 3-fold to favour the carboxylation reaction (Keerberg *et al.*, 2014). Figure 2 describes the cellular location of photorespiration and carboxylation in the differing photosynthetic types. Yet, the influence of photosynthetic type on photorespiratory CO_2_ recovery raises many interesting questions regarding diversity between phenotypes which are answered here by Schlüter *et al.* (2023) through closely examining a range of species in the *Brassicaceae*, which includes several economically important crop species.

**Fig. 1.**
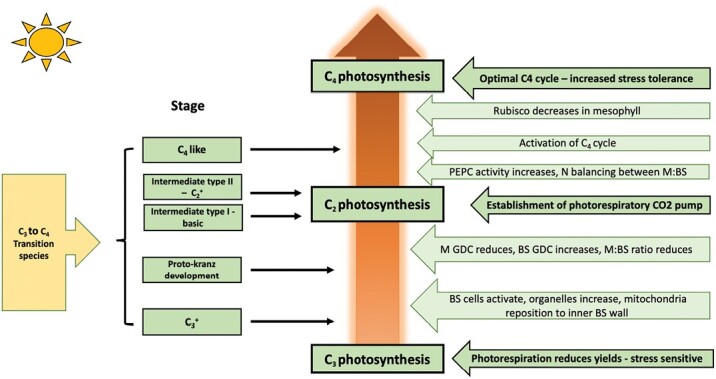
Evolution of C_4_ photosynthesis from the ancestral C_3_ state. C_3_–C_4_ intermediates use a photorespiratory CO_2_ pump (or shuttle), also termed ‘C_2_ photosynthesis’, referring to the two-carbon molecule 2-phosphoglycolate (2PG) produced from O_2_ fixation by Rubisco. 2PG is a toxic molecule that must be processed by photorespiration. Some species remain in a stable C_3_–C_4_ state without progressing further towards C_4_ photosynthesis. Consequently, these species may develop an optimized version of C_2_ photosynthesis, becoming a ‘super C_2_’ in comparison with species that conform to the evolutionary trajectory from C_3_ to C_4_. Each step along the evolutionary continuum requires an orchestration of anatomical and biochemical upgrades to support transitional states. Through identifying these metabolic, anatomical, and physiological markers, the photosynthetic type can be determined. BS, bundle sheath; M, mesophyll; GDC, glycine decarboxylase; PEPC, phospho*enol*pyruvate carboxylase. Adapted from Sage *et al.* (2014).

**Fig. 2.**
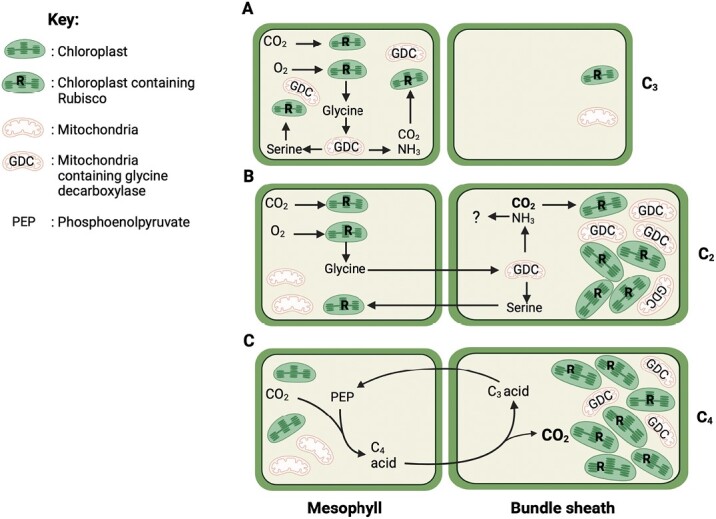
Cellular distribution and anatomical differences of the carboxylation and oxygenation of Rubisco in C_3_, C_3_–C_4_ intermediate, and C_4_ species. In all three photosynthetic types, Rubisco fixes CO_2_ to ultimately enter the Calvin–Benson–Bassham cycle. Most plants use C_3_ photosynthesis (A), where Rubisco also assimilates O_2_ in up to 25% of its reactions, and the toxic product of O_2_ fixation, 2-phosphoglycolate, must be processed by the photorespiratory cycle (Sharkey, 1988). C_3_–C_4_ photosynthesis (or C2) B) exploits photorespiration to realize a simple carbon-concentrating mechanism (CCM) by diffusing glycine to the bundle sheath for decarboxylation (Rawsthorne *et al.*, 1988). Through restricting glycine decarboxylase activity to the bundle sheath, the C_3_–C_4_ intermediates force a division of photorespiration, which increases CO_2_ concentration around Rubisco, at the expense of releasing NH_3_, which must be reassimilated. Consequently, C_3_–C_4_ intermediates can reclaim CO_2_ previously released from photorespiration more effectively in comparison with C_3_ species. In contrast, for C_4_ photosynthesis (C), phospho*enol*pyruvate carboxylase (PEPC) is the primary assimilatory enzyme that lacks affinity for O_2_, producing a C_4_ organic acid to shuttle carbon into the bundle sheath for decarboxylation and raising the concentration of CO_2_ in bundle sheath cells, where Rubisco is located. By favouring the carboxylation reaction of Rubisco, the C_4_ CCM largely suppresses the need for photorespiration, increasing the efficiency of CO_2_ assimilation at the cost of the additional energy needed to regenerate the initial CO_2_ acceptor, phospho*enol*pyruvate (PEP). Anatomical and biochemical indicators show differences between the photosynthetic types to confirm their identity (see also Fig. 3). C_3_–C_4_ intermediates and C_4_ species usually present enlarged bundle sheath cells containing an abundance of organelles that are larger than those seen in C_3_ species, although lower numbers of mitochondria are found in the bundle sheath cells of C_4_ species (Sage *et al.*, 2014). Created with BioRender.com.

## Photosynthetic diversity in *Brassicaceae*

Despite natural occurrence in warm and water-limited environments, which generally favour C_4_ photosynthesis, no members of the *Brassicaceae* use this mode of photosynthesis (Apel *et al.*, 1997). Nevertheless, several C_3_–C_4_ intermediate species in this family have acquired a photorespiratory pump, which is thought to be a pre-requisite for C_4_ evolution (Sage *et al.*, 2012). This suggests that there was potential for evolution of C_4_ photosynthesis in the *Brassicaceae*, so the absence of this mode of photosynthesis and persistence of C_3_–C_4_ intermediacy in some members of the family raises interesting questions. For example, were there other factors that prevented development of C_4_ photosynthesis in the Brassicaceae, or could C_3_–C_4_ intermediacy be the optimal photosynthetic type for the natural environments where C_3_–C_4_ intermediate species occur (Blätke and Bräutigam, 2019)? It should be noted that C_3_–C_4_ intermediacy is remarkably rare; among the 380 000 species of plants, only ~50 C_3_–C_4_ intermediate species have been confirmed to date (Sage *et al.*, 2018). Of course, the true number is likely to be much higher, as many species have not yet been examined in detail and assignment of a given species to a photosynthetic type is not trivial as it requires assessment of multiple physiological and anatomical parameters (Lundgren, 2020). Currently, the only C_3_–C_4_ intermediate species in commercial production is wild rocket (*Diplotaxis tenuifolia*), a member of *Brassicaceae* that includes many important C_3_ crop species (Lundgren, 2020). Schlüter *et al.* (2023) investigate the extent of metabolic variation within *Brassicaceae*, incorporating 75 parameters implicated in photosynthesis to elucidate the diversity present across this intriguing plant family in comparison with their closest C_4_ relative—*Gynandropsis gynandra* from the *Cleomaceae* (a sister family of the *Brassicaceae* in the order Brassicales). In doing so, the authors successfully clarify another case of C_3_–C_4_ intermediacy in *Hirschfeldia incana* (HIR3) within the *Brassicaceae*. HIR3, an accession of *H. incana,* is genetically distinct from both HIR1 and NIJ accessions, appearing closer to the *Sinapis pubescens* lineage as identified by Guerreiro *et al.* (2023), meaning that independent evolution of the photorespiratory shuttle occurred within this lineage.

Additionally, to ease identification barriers, Schlüter *et al.* (2023) propose recording assimilation data at low CO_2_ as their measurements show significant correlations between the CO_2_ compensation point and assimilation rate at 50 ppm CO_2_. Another suggestion by the authors is to use chlorophyll fluorescence measurements of photosynthesis efficiency (*F*_v_/*F*_m_) and measurements of stomatal conductance as diagnostic parameters for assignment of a given species to a photosynthetic category following positive correlation between the net assimilation rate and electron transport rate. These recommendations could reduce time-consuming data collection and allow for quicker confirmation of photosynthetic type for future researchers.

In their investigation, Schlüter *et al.* (2023) examine anatomical, biochemical, and physiological lines of evidence alongside phylogenetic analyses to determine how these parameters interact and their effect on lineage-specific traits. The authors’ results show remarkable diversity across 28 species of *Brassicaceae*, 14 of which were classified as C_3_–C_4_ intermediates. Sequence data from 102 orthogroups revealed the phylogenetic relationship of the species, uncovering examples of convergent evolution across five lineages within the *Brassicaceae*.

The influence of photosynthetic type was found to affect performance under contrasting environmental conditions, with intermediate species showing strengthened CO_2_ assimilation rates under low to subambient CO_2_ concentrations, owing to their CCM, in comparison with C_3_ species. Although the extent of CO_2_ reclamation shows broad variation within a species despite photosynthetic type, previous research in rice shows much intraspecific diversity regarding CO_2_ recapture between wild and cultivated species, indicating improved nitrogen use efficiency for those with more successful CO_2_ recovery rates (Joo *et al.*, 2022).

**Fig. 3.**
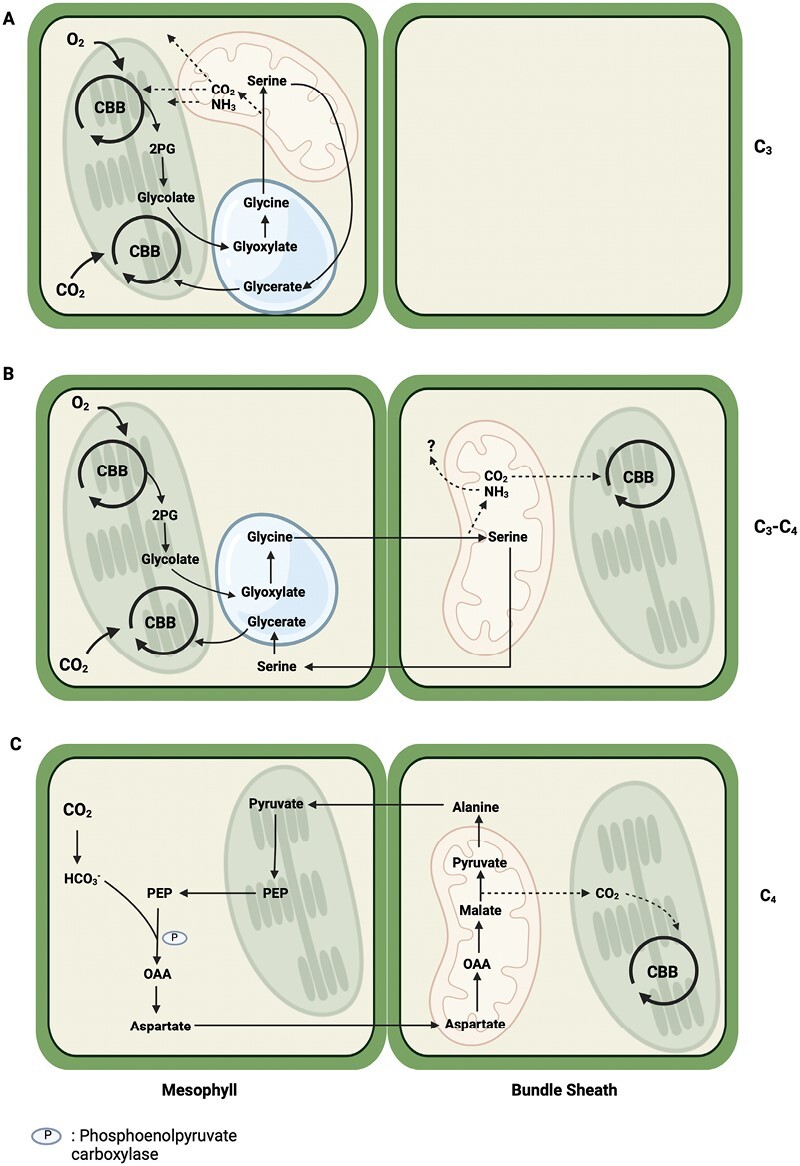
Biochemical dynamics between organelles and cellular localization of oxygenation and carboxylation reactions of Rubisco in three modes of photosynthesis, as described by Schlüter *et al.* (2023). C_3_ photosynthesis (A) keeps both photosynthesis and photorespiration contained within the mesophyll. Photosynthesis occurs in the chloroplast, but peroxisomes and mitochondria are also required for photorespiration to take place. Photorespiration is needed to prevent accumulation of the toxic molecule 2-phosphoglycolate (2PG) in both C_3_ and C_3_–C_4_ metabolism. In C_3_–C_4_ photosynthesis (B), the carboxylation reaction occurs in both the mesophyll and bundle sheath chloroplasts. In contrast to C_3_ photorespiration, the C_3_–C_4_ intermediates diffuse glycine from the mesophyll cell to bundle sheath mitochondria for decarboxylation, thus creating a simple carbon-concentrating mechanism, releasing NH_3_ and shuttling serine back to the mesophyll cell to return to the chloroplast. The process still maintains distribution across the three organelles, similar to C_3_ species, but divides photorespiration across two cell types. The carboxylation reaction of C_4_ photosynthesis (C: NAD-ME subtype) begins with the intake of carbon by phospho*enol*pyruvate carboxylase (PEPC) to form oxaloacetate (OAA) in the mesophyll cell cytosol before shuttling a four-carbon acid (aspartate is shown here) into the bundle sheath mitochondria for decarboxylation. The released CO_2_ is concentrated around Rubisco in the bundle sheath chloroplast and a three-carbon acid is returned to the mesophyll chloroplast before recycling phospho*enol*pyruvate (PEP) into the cytosol for primary carboxylation by PEPC. Photorespiration runs at residual levels in C_4_ species (not shown) owing to the lack of affinity PEPC has for O_2_. CBB, Calvin–Benson–Bassham cycle. Note: (C) denotes the C_4_ subtype NAD-ME present in *Gynandropsis gynandra*, the closest C_4_ relative of the *Brassicaceae*; this species is used as a C_4_ comparison in Schlüter *et al.* (2023). Created with BioRender.com.

The C_4_ pathway of photosynthesis involves initial fixation of CO_2_ in mesophyll cells by phospho*enol*pyruvate carboxylase (PEPC), producing C_4_ acids (malate and aspartate) that move into bundle sheath cells, where they are decarboxylated to release CO_2_ that is refixed by Rubisco. The residual C_3_ moieties are shuttled back to the mesophyll cells in the form of pyruvate, alanine, or phospho*enol*pyruvate to sustain further CO_2_ fixation by PEPC. Figure 2C represents the C_4_ subtype NAD-Me, as used by *Gynandropsis gynandra* in Schlüter *et al.* (2023), in comparison to C_3_ (Fig. 2A) and C_3_-C_4_ intermediate (Fig. 2B) modes of photosynthesis. Additionally, Figure 3 shows biochemical dynamics between organelles within the three photosynthetic types. Metabolite profiles by Borghi *et al.* (2021) examining differing photosynthetic types in the genus *Flaveria* spp. suggested that aspartate might also be shuttled between cell types in C_3_–C_4_ intermediates. Schlüter *et al.* (2023) found no evidence of elevated PEPC activity or C_4_ characteristics of metabolite shuttling in the *Brassicaceae*, but did observe other metabolic and anatomical distinctions between photosynthetic types including multiple amendments that improve the photosynthetic efficiency and potential biological fitness of C_3_–C_4_ species in specific environments. In particular, *D. tenuifolia* exhibited a remarkably low CO_2_ compensation point, almost in line with values usually representative of C_4_ species, meaning that a particularly efficient C_2_ mechanism is present in this species. High levels of malate, aspartate, and glutamate were also found in both *D. tenuifolia* and *Moricandia arvensis* in line with their low CO_2_ compensation points. Analysis of metabolite pools showed much variation within all species, especially for serine levels, in agreement with Fu *et al.* (2023) who found 23–41% variation of serine export under differing photorespiratory conditions in C_3_ species. Under ambient (421 ppm) CO_2_, Schlüter *et al.* (2023) did not find any advantages for the C_3_–C_4_ taxa; however, under ≤200 ppm CO_2_, the intermediate photosynthetic type showed more pronounced water use efficiency than the C_3_ species, suggesting a higher tolerance of drought conditions.

## Climate resilience: future-proof C_3_–C_4_ intermediates

The progression of climate change brings great uncertainty for future food production, alongside an urgent need for solutions to feed an ever-growing global population. Drastic weather fluctuations mean crop species must be resilient to variations in seasonal patterns whilst economizing on resource inputs such as water and nitrogen to improve agricultural sustainability. Future growing seasons are likely to become hotter and drier, on average, and more unpredictable especially in tropical regions, where drought conditions will often be followed by flooding, posing a further risk to crop production. Additionally, by 2050, the atmospheric CO_2_ concentration is expected to rise to 550 ppm, meaning a greening of the planet over the coming years as C_3_ species flourish under high CO_2_ levels owing to a reduction in photorespiratory flux increasing carbon gain (Jaggard *et al.*, 2010). Yet, high CO_2_ concentrations can also instigate stomatal closure, again giving rise to photorespiration, leaving the response of C_3_ species to climate change difficult to foresee. Observations from free air CO_2_ enrichment studies have shown varying effects of elevated CO_2_ on different crop species, with positive effects on photosynthetic activity often being offset by disturbance of C/N balance and source–sink relations (Ainsworth and Long, 2021).

Fortunately, the C_3_–C_4_ intermediates show unique metabolic plasticity compared with other photosynthetic types, meaning that they can switch between C_3_ and C_3_–C_4_ photosynthesis depending on environmental conditions. This capacity provides many advantages, as photorespiration is stimulated not only when stomata close, but also under saline soil conditions, useful for land liable to flooding. Schlüter *et al.* (2023) have shown that C_3_–C_4_ intermediates can not only efficiently reclaim photorespiratory CO_2_ that would otherwise be lost from the leaf, but can also benefit from improved water use efficiency under low CO_2_ conditions that promote photorespiration. Consequently, crop improvement strategies using the C_3_–C_4_ photorespiratory shuttle are likely to be more adaptable to climatic variability and tolerant to abiotic stress in comparison with both C_3_ and C_4_ photosynthetic types. Of course, C_4_ crop species remain productive under hot, dry, conditions, showing good water and nitrogen use efficiencies. However, under extreme weather fluctuations, the benefits of C_4_ metabolism become less apparent, especially under cooler, cloudier conditions.

For *Brassicaceae*, their lack of C_4_ relatives has resulted in a uniquely proficient version of the photorespiratory shuttle in the C_3_–C_4_ intermediates facilitated by an augmented organelle arrangement in the bundle sheath as concluded by Schlüter *et al.* (2023). This anatomical remodelling could also facilitate a non-cyclic mechanism, providing scope for variation within the photorespiratory pathway through an export valve of glycine and serine interacting with other metabolic processes. Given the large number of existing C_3_ crop species within the *Brassicaceae*, this agronomically important family provides ideal candidates for future-proofing food production, both as candidates for domestication and as models for engineering the C_3_–C_4_ mechanism into key C_3_ species to deliver environmental versatility. Schlüter *et al.* (2023) demonstrate not only the broad metabolic diversity present within *Brassicaceae*, but also the means to efficiently identify previously unknown C_3_–C_4_ intermediates in undervalued crop species capable of climate resilience.
